# Biologics in refractory myositis: experience in juvenile vs. adult myositis; part II: emerging biologic and other therapies on the horizon

**DOI:** 10.1186/s12969-019-0361-2

**Published:** 2019-08-20

**Authors:** Anjali Patwardhan, Charles H. Spencer

**Affiliations:** 1 University of Missouri School of Medicine, 400 Keene Street, Columbia, MO 65201 USA; 2 University of Mississippi Medical Center, Batson Children’s Hospital, Rm 289, 2500 North State St, Jackson, MS 39216 USA

**Keywords:** Idiopathic inflammatory myopathies, Dermatomyositis, Polymyositis, Juvenile dermatomyositis, Interferon, Cytokines

## Abstract

The idiopathic inflammatory myopathies (IIM) until recently have been considered a heterogeneous broad group of six autoimmune muscle diseases. Initially, autoantibodies in IIM (including JDM) and CD8+ T cell-induced cytotoxicity (PM and IBM) were the predominant recognized etiopathology mechanisms used to classify myopathies. In the early late 1990’s to 2000’s, evolving understanding of the molecules such as interleukin (IL), tumor necrosis factor (TNF), interferon (IFN), and other cytokines as well as differences in response to therapies, has led IIM researchers to look beyond previous disease mechanisms. For decades the overexpression of Th1- associated cytokines (TNF-α, IFN-γ and IL-12) in the areas of inflammation in skin and muscle in IIM pointed to Th1 as the primary pathway for inflammation in myositis.

However, in the last decade overexpression and elevated level of Th17-associated cytokines (IL-17, IL-22, and IL-6) were identified in the blood and the inflamed muscles of myositis patients. We also do not know how Th1 and Th2 cytokines work differently in diverse hosts, in different concentrations, in different inflammatory milieus, and in the presence or absence of each other or other adhesion/co-stimulatory molecules such as NF-κB. Also, several autoantibodies to intracellular organelles have been identified in myositis.

In this review, we will discuss the most recent advances in IIM research and how that might bring new biologic therapies to market in the next 5–15 years to assist in the care of our most difficult IIM and JDM patients.

## Background

The idiopathic inflammatory myopathies (IIM) were considered a heterogeneous broad group of autoimmune muscle diseases (polymyositis, adult and juvenile dermatomyositis, juvenile polymyositis, inclusion body myositis, necrotizing autoimmune myositis and myositis related to other systemic autoimmune diseases). In the last century, autoantibodies [Immune-Mediated Necrotizing Myopathy (IMNM) and Dermatomyositis (DM)] and CD8+ T cell-induced cytotoxicity [Polymyositis (PM) and Inclusion Body Myositis (IBM)] were the primarily the only two recognized etiopathologies used to classify myopathies [1–3].

We now know that despite similarities in the clinical presentation in these individual subgroups, there are differences at several levels such as infiltrating immune cells, expression of interleukins (IL), tumor necrosis factors (TNF), interferons (IFN), and cytokine profiles as well in response to IIM host response to therapies targeting these molecules. The overexpression of T helper type 1 (Th1)-associated proinflammatory cytokines in the areas of inflammation such as TNF-α, IFN-γ, and IL-12 supported that Th1 might be the primary pathway for inflammation in myositis. Yet lately the recognition of Th17-associated cytokines such as IL-17, IL-22, and IL-6 in the blood and other areas of inflammation in some patients with myositis has challenged our current knowledge on the etiopathology of myositis [4, 5]. These advances may provide new targets for therapeutics especially where the clinical response cannot be achieved by conventional non-targeted immunotherapy such as corticosteroids, immunosuppressive, IVIG, and by available biologic therapies often used in other rheumatic and other inflammatory diseases.

Several autoantibodies have been discovered against intracellular organelles in myositis patients, but we do not entirely understand their pathogenic role or broader interactions in the larger diseases process. The same applies to various cytokines as they have a different effect based on their concentrations, the difference in inflammatory milieus, and the presence or absence of other adhesion co-stimulatory molecules such as nuclear factor NF-κB [6].

The research efforts to unlock the therapeutic unexplored targets in adult myositis patients surpass that in pediatric patients owing to the far lower number of pediatric patients, stricter research regulations for children, and limited funding resources. Thus adult experiences are still extrapolated over for the use in refractory pediatric myositis patients. In part II of this review, we will elaborate on several newly researched treatment options in adult population that are either never been used or have only been used occasionally in the pediatric population.

## Biologics

### Bimagrumab (BYM338)

#### The rationale of the use

This is a fully human recombinant monoclonal anti-ACVR2B activin type 2 receptor antibody that was initially developed to treat muscle volume and strength loss due to any cause. The antibody attaches to activin type II receptors (ActRII) thereby preventing ligation of the specific ligands to the receptors and preventing their activation. It is recognized that the myostatin/ActRII pathway down-regulates skeletal muscle volume and thus inhibition of this pathway should produce significant muscle hypertrophy. This drug is seen as a potential therapeutic option for s-IBM patients.

- Amato et al. hypothesized that signaling of transforming growth factor β superfamily through ActRII, is implicated in the pathophysiology of s-IBM. [7]. They performed a small placebo-controlled study to prove their hypothesis. They recruited 14 patients, 11/14 in a treatment arm and 3/14 in a placebo arm. The patients (14/14) were tested for the inhibition of ActRII after receiving a single dose of Bimagrumab. Most of the patients (12/14) were followed for 16 weeks. The primary outcome measure was to show a change in muscle volume by an MRI of the right thigh muscles at the eight-week point. Secondary outcome measures included muscle strength, function, and lean Body Mass Index (BMI). The treated arm showed a 6.5% increase in right thigh muscle volume as compared to the placebo arm (*p* = 0.024) [7].
- Ioannis et al. performed a literature search and identified four research studies on bimagrumab including one phase III study, and one open-label trial series on alemtuzumab. Although the primary endpoints were not met in the studies, the bimagrumab still showed promising results. Further randomized control trials are needed to know its value conclusively in the treatment of IIM [8].
- A phase IIb/III double-blind, placebo-controlled multicenter study named RESILIENT (supported by Novartis Pharma) is now complete (NCT01925209). A total of 251 s-IBM patients were recruited. The intervention included bimagrumab (10, 3, 1 mg/kg) or placebo every 4 weeks for at least 48 weeks. Patients were evaluated at 52 weeks using the 6-Minute Walking Distance (6MWD) test and the s-IBM- Functional Assessment (sIFA) for muscle strength assessment. The study failed to reach the primary endpoint.
- A follow-up phase three study was performed (July 2015 till February 2017) on patients initially recruited in core study (CBYM338B2203) (NCT02573467) to assess the efficacy, safety, and tolerability after three doses of bimagrumab. They also looked at the long-term effects of bimagrumab. The results are not yet available.

#### Summary

Theoretically, inhibition of the myostatin/ActRII pathway is appealing to achieve significant muscle hypertrophy in atrophied and damaged muscles of myositis patients.

However, neither the available research, nor patient experience is sufficient at this point to establish conclusively its utility in IIM patients. This drug may become valuable for s-IBM patients, but its value in other subgroups and types of myositis has not yet been evaluated.

### Sifalimumab (MEDI-545)

#### The rationale of the use

Sifalimumab is an anti-IFN-α monoclonal antibody. Type I IFN signature, which connects more with IFN-β than IFN-α protein expression, appears to be closely linked with the pathogenesis of adult and pediatric dermatomyositis [9–14]. Various studies using immunohistochemical data established that that Type I IFN is elevated in both the involved and uninvolved muscles of these patients. Their skin and muscle were also infiltrated with plasmacytoid dendritic cells (DC). The serum of the patients with positive anti-Ro/anti-La antibodies and anti-Jo-1 myositis-specific antibodies is known to induce interferon in normal serum [15].

Some researchers in this area believe that measuring type-I IFN-inducible transcripts is a more sensitive and better tool to gauge diseases activity than to measuring serum free IFN-α levels [10, 16–18] as Type I IFN inducible transcripts in the blood of dermatomyositis patients have thus far correlated better with disease activity [19–22].

Clinical Studies:

- The results of the double-blind, phase 1b multicenter randomized control trial (RCT) involving 26 polymyositis and 25 adult dermatomyositis patients, are available. This study (NCT00533091) aimed to evaluate the ability of the anti-IFN-α monoclonal antibody (sifalimumab) to neutralize the type I IFN gene signature (IFNGS) in found in serum as well as in muscles at inception and after the therapy in adult myositis patients. This study looked at the pharmacodynamic and not necessarily a clinical aspect of sifalimumab therapy. The study did achieve its outcome measures, and the results confirmed that sifalimumab therapy significantly reduced the IFNGS in muscles as well as in serum of patients with myositis.

Although this study did not have a clinical outcome measure such as clinical efficacy, clinical improvements were measured using MMT8 on the 98th day of the study. Interestingly, the results showed that the subjects from the placebo as well as treated arm both showed improvements as per the study definition of improvement. The subjects were all adults with myositis and the study power was average [23].

- As a sub-study of the above study, several T-cell related proteins were measured. These T cell-associated proteins included IL-18, TNF receptor 2 (TNFR2) and soluble IL-2RA. The researchers explored if these protein serum markers could be modulated by anti-IFN-α mA therapy. The results showed a significant reduction in the serum T-cell associated proteins in the treated arm vs. placebo arm and concluded that INF blockage could block the T-cell activation which in turn could reduce the T-cell infiltration of muscles in myositis patients. The researchers suggested that change in soluble IL-2RA levels may serve as a biomarker for response to therapy in patients treated with sifalimumab [24].
- Another phase-2, single group assignment, open-label study looking at the long-term tolerability and safety sifalimumab in adult patients with 118 SLE and myositis diagnoses was completed in 2016 (NCT00979654). Out of 118 patients screened, only 103 patients who fulfilled the criteria were recruited, 67/103 completed the study, and 36/103 withdrew because of side effects or other reasons, and one death was reported. A total of 101/103 of Treatment Emergent Adverse Events (TEAEs) and 27/103 Treatment Emergent Serious Adverse Events (TESAEs) were reported from the drug administration until week 182. A business decision to discontinue further development of the sifalimumab at the time was made. A restrictive agreement between the principal Investigators (PI) and the sponsor disallowed the PI to publish the results of the research outcome but some of that can be looked at the Clinical Trials website (NCT00979654).
- Sifalimumab blocked the IFNGS in blood and muscle tissue in myositis patients. A correlation between the IFNGS nullification and improvement in diseases activity has been reported in patients treated with sifalimumab [23].

#### Summary

There is definitive evidence that interferon plays a significant role in the etiopathogenesis of JDM and DM. The above studies suggest that sifalimumab may prove effective and safe in DM in adults. The safety and efficacy of sifalimumab in pediatric myositis patients has not yet been evaluated though trials are anticipated.

Polymyositis may not only have a different etiopathogenesis than DM/JDM, but it is also a very heterogenous group of diseases as PM is often used as an umbrella term to include all non-specific myositis patients [21]. Yet it is still believed that interferon plays some role in even PM. These observations will require further multicenter studies in adults and children.

### Siponimod

#### The rationale for the use

Siponimod is an oral selective sphingosine-1-phosphate receptor (S1P family) modulator (code name BAF312). Siponimod binds selectively to sphingosine-1-phosphate receptor 1 and a few more similar receptors on the lymphocytes and immune cells. Through sphingosine 1-phosphate receptor immunomodulation, 80% of T-cells get trapped in the peripheral lymphoid organs. This binding also inhibits the migration of lymphocytes and other immune cells to the areas of inflammation, thereby reducing inflammation. These effects suggest the rationale of the use of siponimod in active dermatomyositis.

Clinical Studies

- A multicenter, phase 2, double-blind, randomized, controlled trial (NCT02029274) was conducted to assess clinical efficacy and dose response of siponimod in adult (age 18–70 years) dermatomyositis patients who had failed or were intolerant to conventional therapy. In the study, 18 patients with adult dermatomyositis were recruited, and 16/18 received siponimod. The trial was completed in February 2016. The International Myositis Assessment Study (IMACS) definition of improvement was used. The peripheral blood absolute lymphocyte count was used to monitor drug pharmacodynamics (PD). The siponimod was well tolerated, and no significant side effects were reported in the observations. Unfortunately, the ineffectiveness of BAF312 in active DM was recognized early in the study and therefore the study was prematurely terminated.
- A similar study on 29 patients which involved DM as well as PM was conducted.
  Unfortunately, this study was also prematurely terminated (2012). Though, the observations of this study were submitted to clinical trials.gov (October 2018) but are not yet publicly available. (NCT01148810).

#### Summary

So far in two research trials, siponimod is not found to be effective in the treatment of active IIM. Results are not available in the public domain from the second trial in which patients with both DM as well as PM were recruited. The safety and efficacy of siponimod has not been studied in juvenile myositis.

### Apremilast

#### The rationale of the use

Apremilast is a phosphodiesterase-4 (PDE-4) inhibitor. Apremilast increases the cyclic adenosine monophosphate leading to decreased expression of proinflammatory cytokines. It is used successfully in patients with psoriasis, Bechet’s disease and other skin diseases but its exact mechanism of action in myositis is not precisely known. It is hypothesized that apremilast interferes with TH1 and TH2 pathways that play key pathogenetic roles in IIM [25, 26].

Clinical Studies:

- A very small open-label, single-center study with a group assignment from a clinical practice was performed from 2010 to 2015. The primary endpoint was to evaluate the safety and efficacy of apremilast in refractory adult dermatomyositis skin disease patients (NCT01140503). The research period included 12 weeks of therapy (apremilast 20 mg PO BID), and 4 weeks follow-up for a total of 16 weeks. At least 30% reduction in the cutaneous disease activity and severity index (CDASI) (at the baseline and 12 weeks) were the two secondary outcome measures. All patients were between 18 and 65 years old and white females
  Only five patients could be recruited and one patient withdrew. Because of poor recruitment and other technical difficulties, the study was prematurely terminated. Only 1/5 patient reached the threshold of achieving a 30% reduction in the CDASI while 4/5 patients had a mean change in CDASI-activity at 12 Weeks.
  Unfortunately, the observations collected were considered ‘unreliable and ‘uninterpretable’ due to measuring errors. A total of 16 mild adverse events was reported in the 12-week period. No serious adverse effects or fatality were observed [27].
- Bitar et al. reported three patients treated with apremilast. One was a 57-year-old woman with steroid dependent refractory skin-predominant DM (CDASI score 43). The patient had failed several DMARDs as well as rituximab and had developed drug-induced diabetes. The second patient was 65 years old woman skin refractory DM (CDASI score 41). The third patient was 62 years old woman with classic DM with a CDASIc score of 62. All the three cases were biopsy confirmed DM and malignancy had been excluded. All the three patients were started on apremilast (30 mg twice daily) as add on therapy while their steroids and DMARD doses were kept stable. They were rechecked at 1 month after the start and then every 3 months. The apremilast was tolerated well by the patients and no side effects were reported. All three patients showed clinical improvement and a steroid sparing effect starting at 1 month into therapy and at 3 months their CDASI scores were zero, seven and 18 respectively. All the three patients could be weaned off immunotherapy and steroids and maintained on apremilast monotherapy without any relapses [28].
- Another phase two, open-label, single group assignment, interventional study (2018–2019, NCT03529955) is recruiting currently for recalcitrant cutaneous DM patients. The study is assessing safety and efficacy of apremilast (30 mg twice daily) and clinical response at 1 month and 3 months from the initiation of the study. The estimated enrollment is 10 patients. The sustainability of the response will be assessed at the six-month visit [29].

#### Summary

Apremilast has been evaluated to be used in refractory cutaneous DM as an adjunctive therapy and has shown promising safety and tolerability as well as reasonable efficacy in a very limited pool of adult refractory skin disease DM patients. Better quality research and clinical experience are needed to make conclusive recommendations. The long-term efficacy and safety of apremilast as an adjunctive therapy in patients with recalcitrant cutaneous DM also bears more study. More importantly, the efficacy of apremilast has also not been proven in muscle disease in adult DM patients. As expected, there are no reports yet of efficacy and safety of apremilast in childhood myositis.

### Gevokizumab

#### The rationale of the use

Increased levels of IL-1β have been identified in multiple autoimmune diseases such as type I diabetes, rheumatoid arthritis, and autoinflammatory diseases. The interleukin-1-beta (IL-1β) is a pro-inflammatory cytokine with a critical role in the innate immune response in humans. Gevokizumab is a humanized IgG2 monoclonal antibody against human IL-1β with a long half-life that allows once a month therapy. Gevokizumab blocks the interleukin-1 beta (IL-1 beta) and can down-regulate the inflammation that leads to cellular signaling. It is also considered to work through ‘allosteric modulating’. It appears to be a therapeutic candidate for a wide range of immune inflammatory diseases.

Clinical Studies:

- A proof-of-concept, randomized, double-blind, placebo-controlled trial was started (2013–2015) to look at the efficacy of gevokizumab (60 mg SC every 4 weeks for 24 weeks) in 27 adult IIM patients (age ≥ 18 to 65 years) who were recruited in multiple centers in Europe (EudraCT number: 2012–005772-34). Unfortunately, not only the study was prematurely terminated due to financial reasons, the technical and measurement errors were also identified which made the collected observations unreliable.

#### Summary

Studies are very preliminary and thus gevokizumab is not currently recommended as a treatment option for refractory IIM. Yet the biologic may yet have some promise for IIM. As expected, the safety and efficacy of gevokizumab have not been investigated in juvenile myositis.

### Eculizumab (h5G1.1-mAb)

#### The rationale for the use

Eculizumab is a monoclonal humanized antibody against terminal complement components. It inhibits cleavage of C5 to its components (C5a and C5b-9) and may reduce or prevent inflammation in JDM/DM. A single dose of 8 mg/kg of eculizumab has shown to suppress complement activity in the serum in patients with non-myositis complement-mediated diseases. Thus, activation of the complements is considered the final common pathway leading to vascular inflammation and ischemic muscle damage in JDM/DM. The FDA has given the orphan drug status to eculizumab for the treatment of patients with DM in adults (2000, [30]).

Clinical Studies:

- In a randomized, double-blind, placebo-controlled pilot study in DM patients, treatment with eculizumab did improve global physician scores for cutaneous diseases. This study involved the total of 13 DM patients with 10 in the treatment arm and 3 in the placebo arm. The study period was 8 weeks. No serious adverse effects were noted, and the incidences of minor adverse effects were the same in treated and placebo arm. The improvement in MMT (6%), physician global score (9%) and in skin disease score (37%) from the baseline was reported in the treatment arm. The placebo arm showed worsening [31, 32].
- Stanislas Faguer et al. (2018) reported successful treatment with eculizumab of a 19 years old refractory DM patient with thrombotic microangiopathy (TMA) as a comorbidity [33].
- A phase two, randomized, placebo-controlled, third-party-blind study evaluating the safety and efficacy of eculizumab in 17 adult DM patients was recently been completed (2000–2001, NCT00005571) but the results have not been reported [34].

#### Summary

One study for the efficacy of eculizumab in IIM suggests benefit. More need to be done. The biologic has not been investigated so far in juvenile myositis.

### Basiliximab

#### The rationale for the use

Basiliximab is an interleukin-2 receptor (IL-2R; CD25) chimeric monoclonal antibody that binds to IL-2 receptor on the activated T cells. The expression of interleukin − 2 receptor-α (IL-2Rα, or CD25) is especially upregulated on activated T and B cells. A small amount of IL-2Rα is also present in ordinary healthy people on inactive T and B cells and serum as soluble IL-2 receptor (sIL-2R). The increase in the expression of IL-2Rα, as well as sIL-2R, occurs in autoimmune diseases. One rationale for basiliximab use in myositis is that sIL-2R is correlated with disease activity in some DM/JDM patients. This finding also supports the hypothesis that activated lymphocytes play a role in the pathogenesis of DM/JDM/PM.

Clinical Studies

- Jing Zou et al. reported a case series of four adult amyopathic DM patients (positiveanti-MDA5 antibody) who had failed conventional therapy. Three of 4 patients with rapidly progressing ILD demonstrated improved survival, reduction in ferritin levels, and improved lung functions in with use of two doses of 20 mg IV basiliximab 7 days apart [35].
- An open-label, randomized, parallel assignment without masking, phase-2, single center study in China is currently enrolling. This study will be looking at the safety and efficacy of basiliximab as an adjunct treatment in amyopathic dermatomyositis (CADM) patients with interstitial pneumonia. The study duration is to be 52 weeks, and the target recruitment is 100 patients 18 years to 65 years old (NCT03192657). The primary outcome measure is survival at 52 weeks [36].
- The secondary outcome measures are forced vital capacity, total lung capacity and diffusing capacity, serum ferritin, serum KL-6, and semi-quantitatively assessed lung CT change at 52 weeks. The start date was to be July 2017, and the expected end date is June 2020, but as per clinical trials.gov website, they are not yet recruiting [36].

#### Summary

Basiliximab may offer a promising therapeutic option for amyopathic DM patients with rapidly progressive ILD. It has not been tried in other IIM diseases including JDM. Thus, it may be very useful in IIM and JDM in the next decade.

## Emerging therapies

### Anti–T-lymphocyte immunoglobulin (ATG)

#### Rationale for its use

The ATGs treatment causes increase in the percentage of subsets of CDCD25 + Foxp3+ regulatory T cells (Tregs) and depletion of autoreactive T cells. This leads to the gaining regulatory immune cell functions and long-term immunomodulation [37].

ATG is also used as co-therapy in refractory myositis, SLE, scleroderma and other refractory chronic autoimmune disease patients who are selected for stem cell transplant.

Clinical Studies:

- Lindberg et al. performed an open-label randomized single-center comparative study on 11 s-IBM patients, but only 10 patients completed the 12 months follow- up period. The primary goal of this study was to evaluate the safety and efficacy of ATG therapy in s-IBM [38]. The first group (6/11) of patients received oral methotrexate alone (7.5 mg/week for 12 months, the second group (5/11) received IV ATG for 7 days followed by methotrexate for 12 months. The ATG group showed an increase in mean muscle strength by 1.4% as compared to the methotrexate alone group in which muscle strength declined by 11.1% (*p* = 0.021).

#### Summary

The usefulness of ATG therapy in refractory DM/PM myositis patients is unclear though some muscle strength improvements have been reported in IBM patients [38].

### ACTH analogs

#### The rationale for the use

Adrenocorticotropic hormone (ACTH) is a melanocortin peptide. The melanocortin peptide includes α-, vβ-, and γ-melanocyte-stimulating hormone as well as the adrenocorticotropic hormone. There are five types of melanocortin receptors (MC1-MC5) that are known to be expressed in immune cells, muscle cells, podocytes, and glial cells. The melanocortin receptors system is a one of the natural modulatory mechanisms in human beings. These receptors can be stimulated by synthetic ligands and can lead to significant anti-inflammatory and immunomodulatory effects. The adrenal stimulatory and pigmentary effects of ACTH has been known for many years but recent research advances revealed other beneficial effects of melanocortin peptides. Melanocortin has shown to have anti-cytokine properties, and they are known to inhibit inflammatory and immune cell migration into the areas of inflammation [39]. Melanocortin can also influence the autonomic nervous system, exocrine function as well as modulate inflammation, fever, and exocrine secretions and even has antimicrobial effects [40]. Melanocortin can affect inflammation through its steroid-dependent and also through its steroid-independent actions. The gelatin bound purified ACTH analog available is repository corticotrophin injection [RCI]. It was first FDA-approved in the US in 1952 and revised in 2010. It is licensed to use in PM/DM [41]. Its gelatin content allows it to provide an extended release with a long half-life. The most commonly used dose of melanocortin peptide in treatment cycle in. adult myositis patients is RCI 40–80 IU twice weekly SQ for 12 weeks.

#### Clinical trials

There is limited and patchy research on the use of ACTH analogs in adult and childhood myositis.

- Aarat Patel et al. reported their experience with 4 steroid-resistant and refractory myositis patients who were treated with RCI [42]. Malignancy was ruled out in all the four patients. The first patient was a 70-year-old white female with skin biopsy-proven amyopathic DM who failed conventional therapy including rituximab. She was treated with RCI 80 IU SQ twice weekly. She not only showed improvement in her muscle disease but also showed improvement in her bone density after RCI therapy. Unfortunately, her muscle disease relapsed later and a repeat course of rituximab was being considered at the publication of the case series.
  The second case was a 50-year-old white male with electromyography (EMG) and biopsy-proven, skin predominant DM who had failed conventional therapy including steroids and rituximab. He was treated with RCI (80 IU SQ twice weekly) along with azathioprine (AZA) and intravenous immunoglobulin (IVIg). The AZA was later replaced by methotrexate (MTX) as maintenance therapy. He partially responded to RCI, i.e., his muscle enzymes normalized but his severe skin disease continued to flare. The RCI treatment was stopped after 8 months. He later responded to repeat course of rituximab in combination with steroids and hydroxychloroquine. The third patient was a 52-year-old white female with refractory overlap syndrome (PM with Sjogren’s and ILD). She had an inadequate initial response to steroids. She was started on RCI (80 IU SQ twice weekly) as a steroid-sparing agent, before using any biologic agent. Her steroids could be discontinued on RCI therapy, her muscle enzymes returned to normal, and skin lesions improved. Unfortunately, her ILD continued to progress. The rituximab was added to her RCI treatment which appeared to help with her dyspnea. At the time of writing of the case series, her disease was under control on a small dose of oral prednisone, MMF and RCI. The fourth patient was a 57-year-old AA male with an anti-SRP-antibody positive PM who had an initial inadequate response to steroids and later failed conventional therapy including rituximab. The RCI (80 IU twice weekly SQ) was added to his regimen. He responded to RCI, and he could be weaned off his oral steroids. At the time of the publication of the case series, he was asymptomatic, he had normal muscle enzymes and had been on RCI monotherapy for 27 months. The authors stated that the patients did not show exacerbation in their comorbidities during the treatment with RCI. Muscle disease clinical and serologic improvement was seen in all four patients, but skin and lung components of the myositis disease did not appear to respond well. Improvement in the bone density was recorded in one patient and the feeling of general well-being was reported in all the four patients on RCI treatment. Side effects that are commonly seen with steroid therapy, such as hyperglycemia, abnormal weight gain, cushingoid features, hypertension, hyperosmolar states, and skin pigmentation were not reported in patients on RCI treatment.
- Another retrospective report on a case series in which RCI was used in the treatment of biopsy confirmed, refractory PM/DM showed similar results [43]. This case series included five female patients aged 25–68 years with myositis (1 JDM with calcinosis, 2 adult DM, 2 adult PM). All these patients were refractory to conventional therapy but had shown the initial response to steroids. In this series, three out of five patients had also failed rituximab therapy. Four out of five patients in this series were treated with RCI (80 IU twice weekly SQ) while one received only once a week RCI for 12 weeks. Interestingly, they also used RCI to control small flares when continuing on their maintenance treatments. In this series, the RCI was used as a short-term treatment, and therefore, long-term safety and efficacy could not be evaluated. Yet in the short-term, all patients in this series achieved reduced exacerbations over time, increased muscle strength, serologic improvements, reduced skin disease, a decrease in pain, an improved feeling of well-being, and better independent ambulation. Yet since RCI was used as an additive short-term treatment for flares on top of the ongoing maintenance therapy in these patients, any improvements due to RCI could not be conclusively proven, though the effects are very suggestive.

There are differences in these two RCI series. In the first case series reported Above (Patel et al.), the patients had an inadequate response to initial steroid therapy while in the second series (Levine) the patients were steroid sensitive [42, 43]. Also, in the first series the response of skin lesions to RCI was variable while in the second series it was quite good. In the first series, the RCI was used as a part of the main therapy for several months while in second series RCI was used as a short-term bridge therapy to reduce disease load during flares.

- RCI was used with the intention of treatment and evaluation of its safety, efficacy, and steroid-sparing activity in two myositis centers in the USA in an open-label, single group assignment, phase two study (NCT01906372, 2013–2015) on 11 adult refractory myositis (PM and DM) patients. These patients had active disease who failed to respond to steroids and/or one or more immunosuppressive therapy and were defined as refractory. These refractory patients received RCI (80 IU SC twice a week) for 24 weeks. The International Myositis Assessment and Clinical Studies (IMACS) criteria for improvement were used to assess primary endpoint. The safety, tolerability, and steroid-sparing effect were assessed as secondary endpoints. The 10/11 patients completed the study, the median time to reach primary endpoint was 8 weeks in 7/10 patients, and a significant steroid-sparing effect was objectively reported (*P* < 0.01). The RCI was tolerated well, and no significant side effects (infections, hyperglycemia, hypertension, abnormal weight gain) were reported [44].
- There is an ongoing treatment, open-label, phase 4, single group assignment, single-center study (2014–2019, NCT02245841) with an estimated enrollment of 15 refractory cutaneous DM/JDM/ADM subjects (age 18 or above and meet the Bohan and Peter or Sontheimer’s diagnostic criteria). All patients will receive Acthar (80 IU SC twice a week) for 24 weeks. The study is expected to finish by the end of the year 2019.
- An interim observational case study (NCT01637064) was designed to develop a registry aiming to collect long-term experience data on RCI use in refractory PM/DM patients. The primary outcome measure was to look at the safety profile and efficacy of RCI in refractory adult PM/DM patients for at least a year post-treatment. The secondary outcome measures are to see the response in all the subgroups/types of myositis patients with an intent to identify if a particular subgroup of patients responded better to RCI therapy than others [45, 46].

RCT was called Acthar in dermatomyositis and polymyositis treatment (ADAPT) registry as it is supported by Mallinckrodt [46].

The registry includes 25 patients (9/25 DM and 16/25 PM), the median age of 58 years, and malignancies were excluded. Fifteen of 25 were females. The mean time since diagnosis was 3.2 years. Each patient thus far had been treated with a mean of 3.4 drugs before starting on RCI. All of these patients received 80 units of RCI subcutaneously (SC) twice a week. Some patients were on maintenance therapy of 40 units of RCI/week. Side effects thus far only included worsening of glycemic control in 3/25 patients and pedal edema in 2/25 patients. If patients responded, the significant response was observed by or after 90 days in treatment suggesting that patients who discontinued the treatment early in the course may not have had time to respond. Also, it suggests that RCI may have a long latent period for myositis response. The registry is aiming to enroll 100 patients and be able to get 12 months of post-treatment follow-up on these patients.

The weaknesses of this study include that it is not a double-blind controlled RCT, most patients enrolled are females, and no standardization of treatment regimens or washout periods were possible as it is an observational study. Although it is the most extensive observation study for this drug, the number of enrolled patients remains small.

- In an interesting single center, observational ongoing phase 2 trial in which the recruitment is through invitation (2017–2019, NCT03414086). Researchers aim to look at the predictors of clinical response to Acthar in 20 subjects. The researchers are trying to look at the effect of the Acthar at the cellular and molecular level by collecting serum, white cells, pax gene samples and RNA samples from 10 previously Acthar-treated refractory patients (4 PM, 6 DM) from previous research studies and 10 healthy controls. Active myositis patients are not enrolled in this study, but rather patients in remission who were previously treated with Acthar are being recruited along with healthy controls. The study is still in progress.

#### Summary

RCI studies have produced only a few insufficiently designed and powered studies although they have shown limited improvement in muscle as well as the cutaneous aspect of adult myositis so far. Observation periods are of short duration with a maximum length of observation of 1 year. At this point, no data can delineate which specific group or subgroup of myositis patients respond better to RCI than others. No research experience is available on childhood myositis. There have been a few studies’ in childhood myositis patients who were adults at the time of recruitment were included for observation.

There is some suggestion from research experience so far that steroid-resistant patients may show improved response possibly due to immune modulation. Also, RCI therapy does show the steroid-sparing effect in both steroid resistant and steroid-dependent myositis patients. The safety profile of the RCI had been impressive so far, and it is tolerated well with minimal or no corticosteroid side effects such as hyperglycemia, abnormal weight gain, hypertension, and increased infections.

## Conclusions

One difficulty of reviewing potential biologic and other treatments for IIM in the future is that the evidence base is very limited, especially for children. Much can be made of the biologic rationales for the therapies and the excitement for the future is genuine. But most of these treatments may be 5–10 years away from the clinician’s repertoire and patient treatment, if not more. The fate of the drugs and their cost is anyone’s guess.

With that qualification, we are optimistic about:

- Bimagrumab to increase muscle mass in atrophied IIM muscles
- Sifalimumab for all inflammatory myositis diseases due to importance of interferon in IIM.
- Basilixumab for IIM amyopathic disease with interstitial pneumonitis, if not other things as well.
- Eculizumab or Apremilast for cutaneous predominant IIM
- Gevokizumab has theoretical usefulness as an anti-IL-1β therapy in IIM but is untested.
- ACTH analogs appear to be effective in some IIM patients and side effects are surprisingly mild to moderate thus far.

Of this group, sifalimumab must currently be considered the biologic with the most potential in the future due to the strong basic research evidence that interferon is essential to IIM disease etiopathogenesis.

## Data Availability

Is available.

## Abbreviations

ACTH: Adrenocorticotropic hormone
DM: Dermatomyositis
IBM: Inclusion Body Myositis
IFNGS: Interferon gene signature
IIM: Idiopathic Inflammatory Myopathies
IL: Interleukins
IMNM: Immune-Mediated Necrotizing Myopathy
INF: Interferons
JDM: Juvenile Dermatomyositis
JPM: Juvenile Polymyositis
PM: Polymyositis
TNF: Tumor necrosis factors
